# Improved statistical methods for estimating infestation rates in quarantine research when hosts are naturally infested

**DOI:** 10.1093/jee/toad203

**Published:** 2023-10-31

**Authors:** Carole Wright, Pauline Wyatt, David Mayer, Peter Leach

**Affiliations:** Department of Agriculture and Fisheries, Mareeba, QLD 4880, Australia; Department of Agriculture and Fisheries, Ecosciences Precinct, Dutton Park, QLD 4102, Australia; Department of Agriculture and Fisheries, Ecosciences Precinct, Dutton Park, QLD 4102, Australia; Department of Agriculture and Fisheries, Cairns, QLD 4870, Australia

**Keywords:** infestation rate, treatment efficacy, phytosanitary, confidence interval, natural infestation

## Abstract

Trading partners often require phytosanitary or quarantine treatments for fresh horticultural produce to ensure no economically important pest species are moved with the imported product. When developing such treatments, it is essential that the level of treatment efficacy can be determined. This is often based on the mortality of the total number of target pests exposed to treatment, but in naturally infested products this number is not always known. In such cases, the infestation rate and subsequently an estimate of the number of pests are obtained directly from a set of untreated control samples of the host product. The International Plant Protection Convention (IPPC) Secretariat has provided 2 formulas for these situations that place an interval around the point estimate obtained from the control samples to obtain an estimate of the infestation rate. However, these formulas do not allow a confidence level to be assigned to the estimate, and there are concerns with the assumptions regarding the distribution and the measure of variability used in the formulas. In this article, we propose 2 alternative formulas. We propose that the lower one-sided confidence limit should be applied to all infestation datasets that are approximately normally distributed. As infestation data are sometimes skewed, it is proposed the lower one-sided modified Cox confidence limit is applied to data approximately log-normal distributed. These well-recognized formulas are compared to the formulas recommended by the IPPC and applied to 3 datasets involving natural infestation.

## Introduction

International trade of horticultural produce is supported by providing confidence that produce is free from pests of quarantine concern. To provide that confidence, phytosanitary or quarantine treatments are often required, with trading partners often needing evidence of treatment efficacy through data generated according to internationally accepted research guidelines. Treatment schedules for fresh produce moving between jurisdictions, include cold storage, fumigation, irradiation, or heat treatments such as water, air, or vapor (Follett and Neven 2006, Dohino et al. 2017, Moore and Manrakhan 2022). When developing new schedules (dose, temperature, and duration) for these phytosanitary treatments where they do not already exist for a particular pest/commodity/country, testing on sufficient numbers of the target pest on or in the host is required to demonstrate an acceptable level of treatment efficacy. Treatment efficacy testing is often based on the mortality of the target pests in the host produce, meaning the infestation rate pretreatment and counts of mortality posttreatment are critical parameters in determining efficacy. Other measures of efficacy include sterility, or the inability to fly or complete development (USDA 2016).

Historically, it has been a requirement that before a treatment schedule can be accepted, a minimum of either 30,000 or 93,616 target insects are treated with no survivors. Treating 30,000 target insects equates to 95% confidence that the lower bound of mortality is at least 99.99% (probit 8.7). In comparison, treating 93,616 insects with no survivors equates to 95% confidence that the lower bound of mortality is at least 99.9968% (probit 9). Probit 9 was first proposed by Baker in 1939 (Baker 1939) and has been adopted as an unofficial standard by some importing countries, while others have accepted the less stringent probit 8.7 (Heather and Hallman 2008).

When determining treatment efficacy, it is not always possible to know the number of insects treated within the host produce due to not knowing, or accurately predicting, the infestation rate pretreatment. This can occur when the host samples are naturally infested; a process whereby the host samples are exposed to mature insect pests which then have a natural instinct to oviposit, depositing an unknown number of eggs into each host sample (Sharp and Hallman 1992, Heather et al. 1997). Natural infestation more closely represents natural conditions and the relationship between host and species compared to artificial infestation. Artificial infestation involves either placing a known number of insects into the host sample (Mangan et al. 1998, Lin et al. 2020) or pipetting a measured aliquot of liquid in which the insects are suspended into the host (Grout et al. 2011, Gazit et al. 2014). If it is determined or known that the method of infestation (natural or artificial) does not affect treatment efficacy, samples can be artificially infested. Using artificial infestation, the insect life stage and infestation rate can be controlled, and hosts (whether good or poor) can be adequately infested before treatment (Hallman 2014). Natural infestation, although better replicating real-world infestation, provides a challenge in that the infestation rate and number of insects treated are not always known, and results in high variance due to the uneven pest counts in each individual host sample (Heather and Hallman 2008). The ability to account for high variability therefore becomes paramount in determining treatment efficacy and needs appropriate statistical support. Furthermore, although samples used in studies developing phytosanitary treatment schedules should be of a quality similar to those used in a commercial setting, variability can arise through subtle differences in host quality which may affect attractiveness for oviposition between host samples. These differences may include visual or olfactory cues, such as chemical composition in surface waxes or of the interior of the host (Díaz-Fleischer et al. 1999) which are not detectable by humans.

After infestation, the host samples are treated using the proposed schedule once insects develop to the appropriate life stage. Posttreatment the host samples are either held to allow insect survivors to emerge naturally with counts of surviving larvae, pupae, or adults recorded or dissected to record the number of live and/or dead insects. To further complicate the determination of treatment efficacy, for some insect species or families (such as tephritid flies) it is not possible to count the number of dead for all life stages. For example, when treating host produce infested with third-instar larvae of tephritid flies it is possible to dissect the treated host samples posttreatment and count the number of live and dead instars to obtain the number of insects treated (Myers et al. 2016, Hallman et al. 2019). However, dead eggs, first instars, and early second instars of tephritid flies cannot be readily observed upon visual examination of the host (Hallman et al. 2019). When counting the number of insects from treated hosts is not possible, a set of control host samples that are infested, but not treated, are assessed at the same time (Wadley 1949). Under the normal process of developing a phytosanitary treatment schedule, up to 20% of samples are kept untreated to act as control samples (Heather et al. 1997, Hallman and Thomas 2011). The resulting number of survivors in these untreated control samples is then used to estimate the infestation rate per control sample and subsequently the estimated total number of insects treated within the treated samples.

In the situation where mortality and survival are recorded in the control host samples, an adjustment for natural mortality can be made using Abbott’s formula (Abbott 1925). This then gives an adjusted mortality based on a known number of control insects and the traditional methods of calculating a confidence limit for the unknown survival rate provided by Couey and Chew (1986) can be applied. In contrast, many studies report only the number of survivors in the control samples and mortality is not recorded (Sharp and Hallman 1992, Heather et al. 1997, Jessup et al. 1998). In such cases, the infestation rate is already adjusted for natural mortality and many researchers simply calculate the mean infestation rate of the treated samples to be the same as that of the untreated control samples (Jessup et al. 1998, Heather et al. 2002). This point estimate for the estimated infestation rate based on the control samples is then multiplied by the number of treated samples to estimate the total number of treated insects.

The simplistic approach described above, assumes the control and treated samples have the same point estimate for the estimated infestation rate, however, for control and treated samples that are naturally infested this is unlikely to be true. To address this variability, 2 formulas are provided in the International Plant Protection Convention (IPPC) Procedure Manual for Standard Setting (2022). The formulas provided by the IPPC attach a lower limit to the simple point estimate used by many researchers, thus giving a conservative estimate for the infestation rate. These 2 formulas have appeared in IPPC procedure manuals dating back to at least 2014 (IPPC 2014) and are portrayed as being based on a 95% confidence level. We have several concerns over the validity of the formulas in the IPPC procedure manual. Here we discuss those concerns and propose alternate, well-recognized, and statistically-based calculations that account for the natural variability that is encountered. The recommended and proposed formulas are applied to 3 datasets which all involved host samples being naturally infested.

## Materials and Methods

### Current Recommended IPPC Formulas

The IPPC procedure manual provides 2 formulas to estimate the treated infestation rate per host sample, defined as the number of survivors per treated sample. For clarity, the term infestation rate will be used from this point forward.

When using control samples to estimate the infestation rate, it is recommended that the infestation rate is recorded for each individual sample (IPPC 2022). In such cases, the IPPC procedure manual (IPPC 2022) recommends the estimated infestation rate per sample be calculated as:


$$\begin{array}{l} \mu -STD\times 1.645 \end{array}$$ (1)

where μ is the mean infestation rate per control sample and STD is the associated standard deviation. This formula will be referred to as the IPPC formula (1).

For small commodities, such as blueberries and grapes, it is not always feasible to hold control samples as individuals, especially when treating large numbers of fruit. In such cases, the control samples are held in small groups of multiple samples (e.g., in punnets) and the infestation rate is based on the mean of the grouped samples. The recommended calculation in the IPPC procedure manual (IPPC 2022) when control samples are kept in groups is:


$$\begin{array}{l} \mu -STD\times \sqrt{\left(1+1/r\right)} \end{array}$$ (2)

where *r* is equal to the number of control groups used to estimate the overall mean (μ) and standard deviation (STD) of the control group means. This formula will be referred to as the IPPC formula (2). These 2 formulas are aimed at giving more conservative estimates than the simple point estimate used by many researchers.

### Proposed Calculations for Estimating Infestation Rate

We propose that the lower 95% confidence interval for the mean infestation rate be adopted to replace formulas (1) and (2) to strengthen the statistical validity of the resulting estimate. The lower limit for a one-sided 95% confidence interval is:


$$\begin{array}{l} \hat{\mu }-STD/\sqrt{r}\times t_{0.05,r-1} \end{array}$$ (3)

where $\hat{\mu }$ is the estimate of the overall mean infestation rate and *t*_0.05,*r*−1_ is the 95th percentile of the Student’s *t*-distribution with *r*−1 degrees of freedom. This formula will be referred to as formula (3) and is appropriate for both individual control samples and grouped samples when the infestation rates are consistent with a normal distribution. In formula (3), $STD/\sqrt{r}$ is the standard error of the mean. For large sample sizes that are not consistent with a normal distribution, the central limit theorem can be applied and the resulting confidence limit estimates using formula (3) remain applicable.

The distribution of the infestation rates may not be approximated by a normal distribution, but be positively skewed, and in these instances can be modeled by a log-normal distribution. For such datasets, the lower one-sided 95% modified Cox confidence limit is suitable (Olsson 2005). The infestation rates are first log_e_ transformed and the sample mean $\left(\bar{Y}\right)$ and variance (*s*^2^) of the transformed data are calculated. These values are then used in formula (4) to calculate the lower one-sided 95% modified Cox confidence limit.


$$\begin{array}{l} \bar{Y}+s^{2}/2-t_{r-1\left(0.05\right)}\sqrt{s^{2}/r+s^{4}/\left(2\left(r-1\right)\right)} \end{array}$$ (4)

It is important to note that the log-normal distribution requires all values to be positive (>0). In the case of small samples that follow non-normal distributions other than the log-normal, more sophisticated methods will be required. This issue can be avoided by ensuring there are sufficient control samples to enable the use of the central limit theorem.

### Application

Three datasets were selected to explore the application of the recommended and proposed formulas described above. All 3 datasets are trials involving natural infestation and the infestation rate was based on the number of survivors in a set of untreated control samples.

### Mango

Organic ‘Kensington Pride’ mangoes (*Mangifera indica* L.) were weighed and placed into 5 batches of 25 fruits of a similar weight. The 25 fruits in each batch were pinholed 20 times to encourage natural oviposition and placed in a 5 × 5 layout in a cage containing *Bactrocera jarvisi* (Tyron) (Diptera:Tephritidae) adult flies which were allowed to oviposit directly into the fruit. Each cage had 5 untreated control samples (20% of all fruit) which were preselected based on a Latin square arrangement. The remaining 20 samples were selected to undergo phytosanitary treatment after infestation. The control samples were held individually after infestation in a controlled environment room set at 26°C and 70%RH and did not undergo any treatment. The number of survivors was recorded for each individual control sample. This dataset was appropriate for the application of the IPPC formula (1) and the lower one-sided 95% confidence interval (formula (3)).

### Capsicum

Organic capsicums (*Capsicum annuum* L.) were weighed and placed into 10 batches containing 25 fruits of a similar weight. Fruit in each batch was punctured 15 times with a pin at the blossom end to encourage natural oviposition before being placed in a 5 × 5 layout in cages containing adult *Bactrocera neohumeralis* (Hardy) (Diptera: Tephritidae). In each cage, 5 of the 25 fruits (20%) were preselected based on a Latin-square arrangement to be the untreated control samples. After infestation, the 5 preselected control samples from a cage were placed in a single container and held in a controlled environment room set at 26°C and 70%RH. The remaining 20 samples were chosen for treatment. The total number of surviving pupae was recorded for each set of 5 control fruits from a cage. IPPC formula (2) and the lower one-sided 95% confidence interval (formula (3)) were appropriate for this dataset.

### Tomato

Naturally ripened organic ‘Daniella’ tomatoes (*Solanum lycopersicum* L.) were punctured 10 times around the flower end and placed in a cage containing adult *Bactrocera tryoni* (Froggatt) (Diptera: Tephritidae) which were allowed to oviposit directly into the fruit. Three cages were used and within each cage fruit were laid out in a 6 × 6 grid, giving a total of 108 samples. All samples can be deemed untreated controls as no postinfestation treatment was applied to any samples. This allowed for the variability of the infestation rate of untreated samples to be explored. After infestation, each fruit was held in an individual container in a controlled environment room set at 26°C and 70%RH to allow the larvae to develop. The number of surviving pupae in each individual fruit was recorded.

Simulations using randomly selected samples to represent the normal 20% quota of control samples were conducted to investigate the range of estimated infestation rates based on the 4 formulas outlined above. A total of 5,000 random subsets of the tomato data were generated, where each subset comprised 6 samples (20%) from each cage to represent the control samples. To follow standard practice when the fruit is laid out in a square configuration when infesting, the 6 randomly selected samples from within a cage were chosen such that they formed a Latin-square arrangement. Each subset was tested to assess if it approximated a normal or log-normal distribution. A likelihood-ratio-based Anderson–Darling goodness-of-fit test (Zhang 2002) was used to assess the approximate distribution of each random subset. Statistical tests were conducted with 0.05 as alpha. As noted earlier, a requirement of the log-normal distribution is that all values must be greater than zero. Therefore, any dataset that contained at least one count of zero infestation rate and did not approximate the normal distribution was automatically considered an alternate non-normal distribution. For random subsets consistent with a normal distribution, formulas (1) and (3) were applied and for those approximated by a log-normal distribution, formulas (1) and (4) were applied. If the random subset could not be approximated by either a normal or log-normal distribution, no estimate of the infestation rate was calculated.

For the 5,000 random subsets comprising 6 samples from each cage, regardless of distribution, the selected fruits within a cage were combined to form 3 groups of control fruit. This created a dataset for which formulas (2) and (3) could be applied.

All simulations and data calculations were performed using Genstat for Windows 22nd edition (VSN International 2022).

## Results

### Mango

The number of survivors from the 25 untreated mango control samples held as individuals ranged from 34 to 318 per host sample (Table 1). Based on the likelihood-ratio-based Anderson–Darling goodness-of-fit test for normality, the infestation rate per sample was sufficiently close to normally distributed (*W* = 3.384; *P* = 0.148). The overall mean infestation rate per control sample was estimated as 132.8, with a standard deviation of 67.45. Applying the recommended IPPC formula (1), which is required when the infestation rate for each individual control sample is known, the estimated infestation rate per sample was 21.9 (≈ 132.8–67.45 × 1.645). In comparison, based on formula (3), the lower one-sided 95% confidence limit gave an estimated infestation rate per sample of 109.8 (≈ 132.8–(67.45/25^1/2^) × 1.711), approximately 5 times larger.

**Table 1.** Infestation rates (number of survivors) of *B. jarvisi* per individual untreated control mango fruit

Fruit no.	Survivors per fruit	Fruit no.	Survivors per fruit	Fruit no.	Survivors per fruit
1	89	10	116	19	176
2	143	11	203	20	118
3	318	12	145	21	54
4	122	13	34	22	83
5	74	14	75	23	98
6	43	15	60	24	199
7	221	16	91	25	160
8	165	17	130		
9	192	18	212		

### Capsicum

For the capsicum trial, control samples were in groups of 5 and the number of survivors per sample within a group ranged from 17.2 to 85.2 (Table 2). The likelihood-ratio-based Anderson–Darling goodness-of-fit test for normality suggests the infestation rate per sample was sufficiently close to normally distributed (*W* = 3.322; *P* = 0.499). The overall mean infestation rate per control sample was 44.8, with a standard deviation of 21.73. Based on 10 control groups and applying the recommended IPPC formula (2), the estimated treated infestation rate was 22.0 (≈ 44.8–21.73 × (1 + 1/10)^1/2^). In comparison, the lower one-sided 95% confidence limit based on formula (3) gave an estimated treated infestation rate per sample of 32.2 (≈ 44.8–(21.73/10^1/2^) × 1.833), approximately 1.5 times larger.

**Table 2.** Infestation rates (number of survivors) of *B. neohumeralis* per untreated control group of 5 capsicum fruits from 10 cage replicates

Cage	Number of control fruit	Number of survivors	Survivors per fruit
1	5	86	17.2
2	5	182	36.4
3	5	145	29.0
4	5	246	49.2
5	5	138	27.6
6	5	302	60.4
7	5	225	45.0
8	5	426	85.2
9	5	136	27.2
10	5	353	70.6

### Tomato

The infestation rate per tomato ranged from 0 to 159, with a median of 43 and a standard deviation of 38.83 (*n* = 108). One fruit in each cage recorded no infestation. Two of these fruit occurred on the outside row of the cage, while the third fruit was in the center.

#### Random subsets approximated by a normal distribution.

**Fig. 1.**
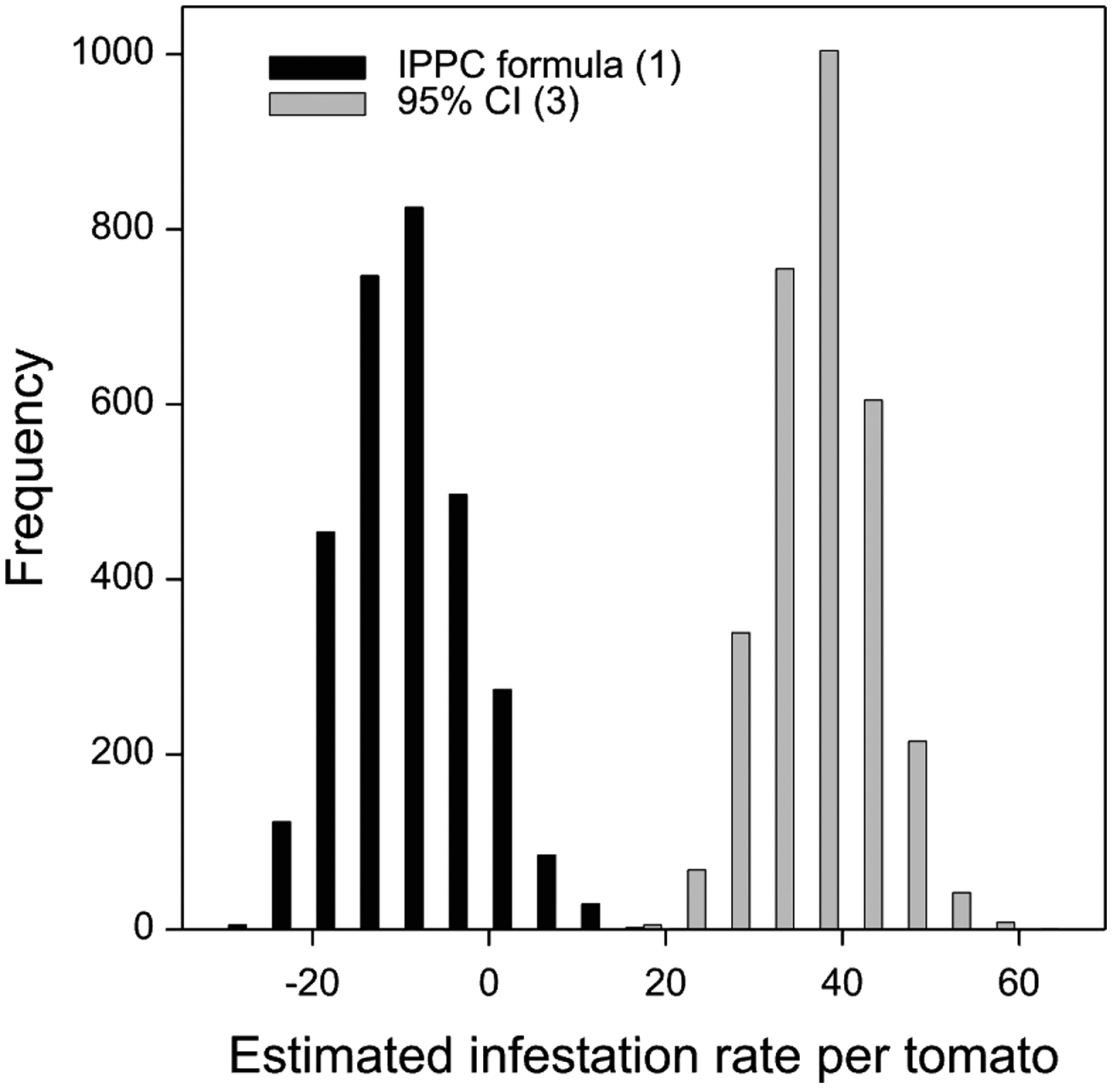
Frequency distribution of estimated infestation rates of *B. tryoni* for 3,042 randomly generated approximately normally distributed tomato control subsets comprising 18 fruits. For each control subset, infestation rates were calculated using IPPC formula (1) (μ−STD×1.645) and the lower one-sided 95% confidence interval (95% CI) (formula (3)) $\left(\hat{\mu }-STD/\sqrt{r}\times t_{0.05,r-1}\right)$. IPPC: International Plant Protection Convention.

Of the 5,000 random subsets with individual control samples, 3,042 (60.8%) were found to sufficiently follow a normal distribution. These subsets were suitable for the application of the IPPC formula (1) and the lower one-sided 95% confidence limit defined by formula (3). The distributions of infestation rates per sample based on IPPC formulas (1) and (3) have very little overlap (Figure 1). The median estimated infestation rate per control sample was negative using the IPPC formula for individual control samples (Table 3). Of the 3,042 subsets, only 391 (12.9%) resulted in an estimated infestation rate greater than zero when using the IPPC formula.

**Table 3.** Summary statistics of estimated infestation rates (number of survivors) of *B. tryoni* per control fruit from a simulation study. Randomly generated subsets of 18 individual tomato control fruit, that were sufficiently close to normally distributed, were subject to IPPC formula (1) (μ−STD×1.645) and the one-sided 95% confidence interval (CI) (formula (3)) $\left(\hat{\mu }-STD/\sqrt{r}\times t_{0.05,r-1}\right)$ and approximately log-normal distributed samples were subject to IPPC formula (1) and the one-sided 95% modified Cox CI (formula (4)) $\left(\bar{Y}+s^{2}/2-t_{r-1\left(0.05\right)}\sqrt{\frac{s^{2}}{r}+\frac{s^{4}}{2\left(r-1\right)}}\right)$. Randomly generated subsets of 3 groups of 6 tomato control fruits were subject to IPPC formula (2) $\left(\mu -STD\times \sqrt{\left(1+1/r\right)}\right)$ and the 95% CI (formula (3)). *N* is the number of randomly generated subsets from which the summary statistics were obtained. IPPC: International Plant Protection Convention

				Estimated infestation rate per control fruit		
No. control fruit	Distribution	Method	*N*	Median	Min	Max
18 individual fruit	Normal	IPPC formula (1)	3,042	–9.0	–27.2	20.8
		95% CI	3,042	36.8	18.4	60.3
	Log-normal	IPPC formula (1)	972	–19.0	–41.0	5.6
		95% modified Cox CI	972	33.1	20.9	53.3
3 groups of 6 fruit	Normal	IPPC formula (2)	5,000	24.7	–7.9	63.4
		95% CI	5,000	13.0	–33.8	61.4

**Fig. 2.**
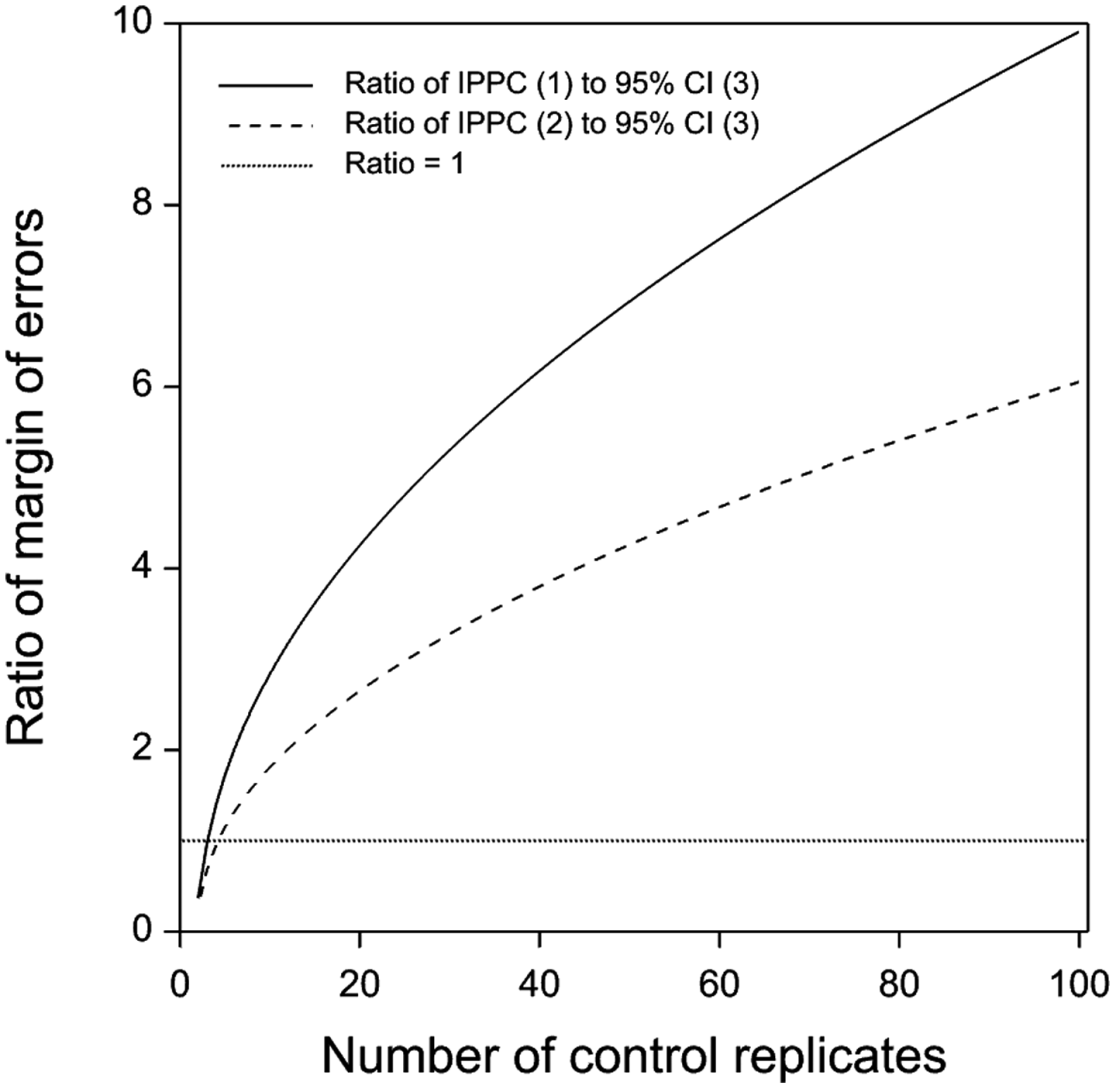
Ratio of margin of errors for recommended IPPC formulas to the 95% confidence interval (CI). IPPC (1) = μ−STD×1.645, IPPC (2) = $\mu -STD\times \sqrt{\left(1+1/r\right)}$, and 95% CI (3) = $\hat{\mu }-STD/\sqrt{r}\times t_{0.05,r-1}$. Horizontal line is equivalence of the 2 methods. IPPC: International Plant Protection Convention.

The ratio of the margin of errors of IPPC formula (1) to the lower one-sided 95% confidence limit given by formula (3) is greater than one when there are more than 3 control samples (Figure 2). Ratios greater than one equate to IPPC formula (1) producing lower estimated infestation rates. With 18 control samples as in this simulation, the ratio of the margin of errors is more than 4.

#### Random subsets approximated by a log-normal distribution.

**Fig. 3.**
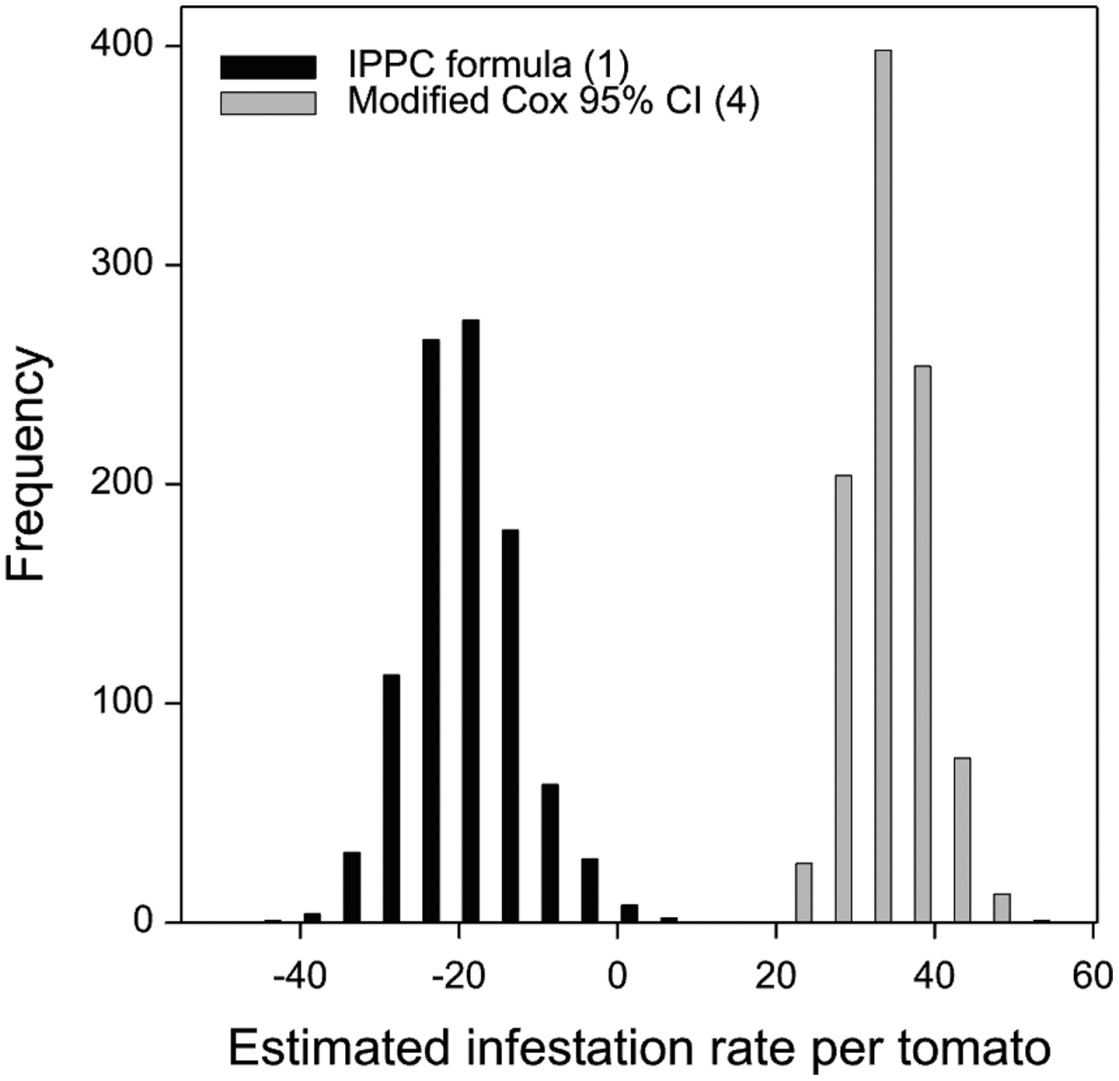
Frequency distribution of estimated infestation rates of *B. tryoni* for 972 randomly generated approximately log-normal distributed tomato control subsets comprising 18 fruits. For each control subset, infestation rates were calculated using IPPC formula (1) (μ−STD×1.645) and the lower one-sided 95% modified Cox confidence limit (formula (4)) $\left(\bar{Y}+s^{2}/2-t_{r-1\left(0.05\right)}\sqrt{\frac{s^{2}}{r}+\frac{s^{4}}{2\left(r-1\right)}}\right)$. IPPC: International Plant Protection Convention.

The IPPC formulas do not discriminate samples based on their distributional properties and therefore formula (1) was again applied to 972 randomly generated subsets that approximated a log-normal distribution when control samples were held as individuals. The distributions of estimated infestation rates based on the IPPC formula (1) and the lower one-sided 95% modified Cox confidence limit (formula (4)) for the 972 random subsets that approximate a log-normal distribution do not overlap (Figure 3). The median infestation rate for IPPC formula (1) was −19.0 compared to 33.1 for the lower one-sided 95% modified Cox confidence limit (Table 3).

#### Random subsets not consistent with normal or log-normal distributions.

There were 986 (19.7%) subsets that did not sufficiently follow either a normal or log-normal distribution when the control samples were held individually. Of these, 920 contained a sample with zero infestation rate and only 66 subsets (1.3%) with positive infestation rates for each individual fruit were not consistent with normal or log-normal distributions. With a sample size of just 18 control samples, the central limit theorem is not applicable.

Of the 5,000 randomly generated subsets, 2,153 selected a sample that had zero pupal recovery and therefore an infestation rate of zero. Based on the properties of the log-normal distribution, these subsets automatically cannot sufficiently follow a log-normal distribution. Of the subsets where a sample with zero infestation rate was selected, 1,233 (57.3%) were found to be sufficiently consistent with a normal distribution.

#### Random subsets of grouped control fruit.

**Fig. 4.**
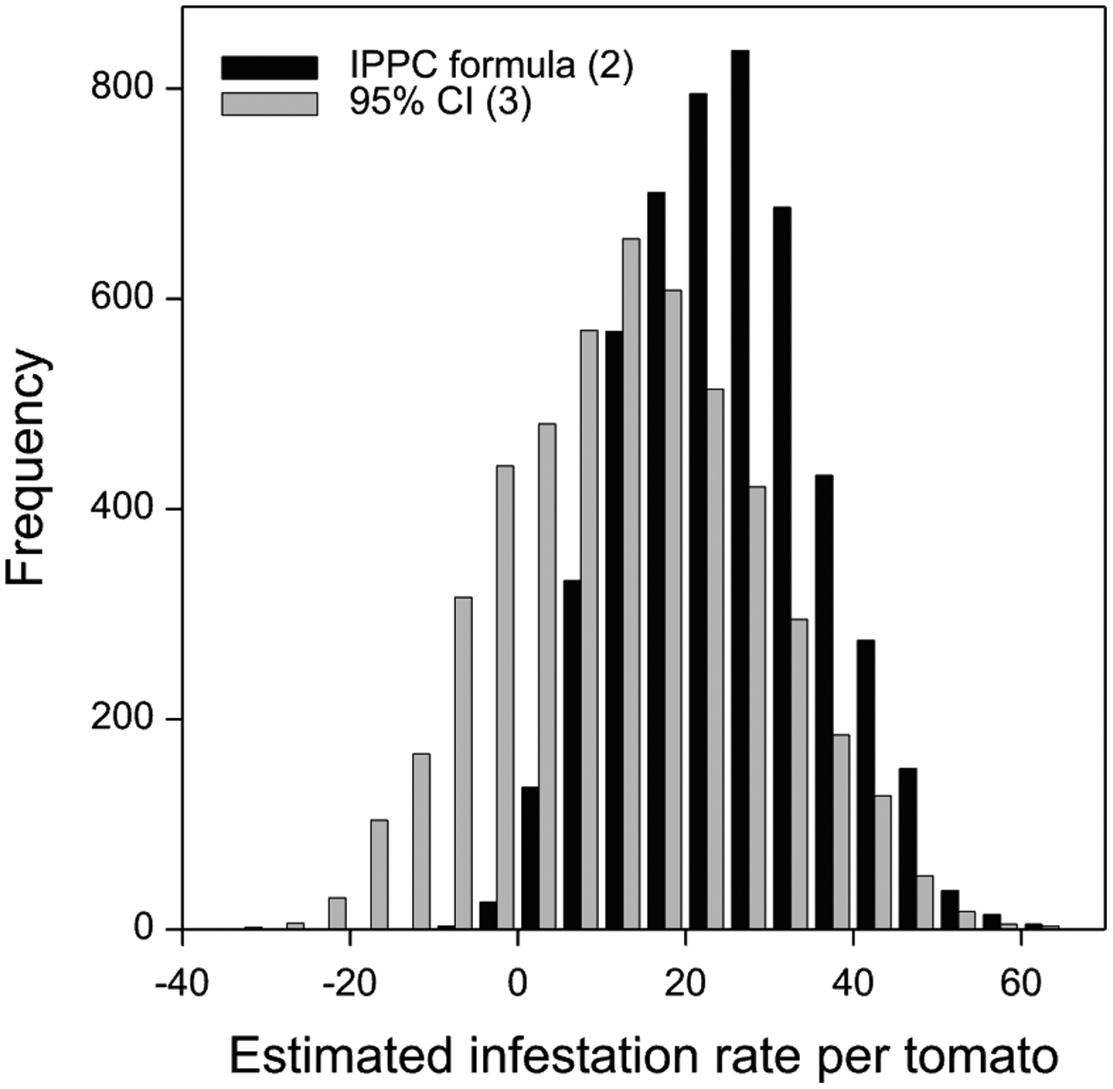
Frequency distribution of estimated infestation rates of *B. tryoni* for 5,000 randomly generated subsets of tomato control fruit in 3 groups of 6 fruits. For each subset of control fruit, infestation rates were calculated using IPPC formula (2) $\left(\mu -STD\times \sqrt{\left(1+1/r\right)}\right)$ and the lower one-sided 95% confidence limit (95% CI) (formula (3)) $\left(\hat{\mu }-STD/\sqrt{r}\times t_{0.05,r-1}\right)$. IPPC: International Plant Protection Convention.

When the pupal recovery counts for the 6 randomly selected samples within the same cage were grouped, an average infestation rate per group was calculated. Based on the average infestation rate for each of the 3 cages, the estimated infestation rate using IPPC formula (2) and the lower one-sided 95% confidence limit (formula (3)) was obtained. The estimated infestation rate per sample for the 5,000 randomly generated subsets with grouped control samples is summarized in Table 3. The distributions of the 5,000 estimated infestation rates significantly overlap for the 2 formulas (Figure 4). The similarity is not unexpected due to the ratio of the margin of errors of IPPC formula (2) to formula (3) being 0.685 when there are 3 control groups (Figure 2).

## Discussion

The application of the formulas to the 3 datasets highlights that different formulas can lead to wide-ranging estimates of the infestation rates and therefore will result in different estimates of the total number of insects treated and ultimately impact the determination of treatment efficacy. It has been shown that the estimates obtained by the IPPC formulas, when applied to samples held individually, as in the mango and tomato datasets, can be excessively conservative. Negative estimated infestation rates occurred for 87.1% of the tomato subsets that were consistent with a normal distribution when using the IPPC formula (1) on individual control samples. These trials would all be declared a failure. Rearranging formula (1) shows that any dataset with a coefficient of variation (CV = STD/$\hat{\mu }$*×*100) greater than 60.79% (= 1/1.645×100), will result in a non-positive estimated infestation rate. This is unlikely to be of concern when using artificial infestation, but natural infestation can be subject to high levels of variability.

When the control samples in the tomato dataset were grouped, the distributions of the estimated infestation rates were more similar. Both the IPPC recommended formula (2) and the lower one-sided 95% confidence limit (formula (3)) produced negative estimated infestation rates. A negative value will be obtained by the lower one-sided 95% confidence limit if the sample CV is greater than *r*^1/2^/*t*_0.05,*r*−1_. With only 3 grouped samples as in the tomato dataset, the 95th percentile of the Student’s t-distribution (*t*_0.05,2_) used in formula (3) is 2.920. Therefore, with only 3 groups, a negative estimate will be obtained if the sample CV is greater than 59.3%. This supports the IPPC recommendation that control samples should be held as individuals to ensure a sufficient control sample size, or alternatively, higher levels of replication of grouped control samples are required when using natural infestation.

Of more importance than the estimates obtained by the formulas, is the statistical theory underlying each formula. The recommended formulas in the IPPC procedure manual have inherent limitations concerning statistical validity. A flaw with the IPPC formula (1) is that it uses the standard deviation as the measure of variability, rather than the standard error of the mean. It is the estimate of the population mean that is being calculated; therefore, the standard error of the mean is a more appropriate measure of the variability (Altman and Bland 2005). As the number of samples increases, the standard error will decrease, but the standard deviation will not tend to change (Altman and Bland 2005). This is reflected in the highly conservative values obtained by the IPPC formula (1) for the mango and tomato datasets. As well as using an inappropriate measure of variability, the IPPC formula (1) provides the researcher with no incentive to increase the number of control samples. It is a common practice by many researchers to have up to 20% of infested pretreatment samples act as untreated control samples. Based on the mean and standard deviation obtained by the mango dataset, to meet the requirement of treating 30,000 target insects, the IPPC formula (1) would require a minimum of 1,713 mango fruits to be infested before treatment if 20% (343 fruits) of these were to act as controls. However, as formula (1) is not influenced by the number of control samples, there is potentially minimal impact on the estimated infestation rate if fewer samples acted as controls. In contrast, the estimated infestation rate calculated by the lower one-sided 95% confidence interval is dependent on the number of control samples. To meet the requirement of treating 30,000 target insects based on the mean and standard deviation for the mango dataset and formula (3), a minimum of 256 mango fruits would need to be exposed to treatment plus a further 64 fruits (20% of the total number of infested samples) acting as untreated controls. If only 10% of the infested samples are to be kept as untreated controls, an additional 14 mango fruits would need to be exposed to treatment post-infestation. Lowering the proportion of control samples will affect the estimated number of treated samples needed to meet the target number of treated pests when using the lower one-sided 95% confidence limit.

Many researchers simply calculate the estimated number of treated insects based on the mean infestation rate of the untreated control samples (Jessup et al. 1998, Heather et al. 2002). This calculation does not consider the variability in the infestation rates. Myers et al. (2016) suggest that uneven infestation when naturally infesting improves the robustness of the results and should not be considered a hindrance. However, Couey and Chew (1986) report that ‘point estimates are *always* wrong’. They recommend the use of an interval surrounding the point estimate which has a level of confidence attached. All 4 formulas are an interval surrounding the point estimate, but only formulas (3) and (4) have a level of confidence attached based on a given sample size. In the IPPC formula (1) there is a reference to 95% confidence through the value of 1.645. This is based on the Student’s *t*-distribution with infinite degrees of freedom at the 0.05 level (*t*_0.05_,_∞_). IPPC formula (1) is thus the lower one-sided 95% confidence limit for the population mean, not for the sample mean.

A related concern with the IPPC formula (2) is that it is not a probabilistic statement. The formula contains no reference to an underlying probability distribution and therefore no level of confidence can be associated with the resulting estimate. If a 99% (or other) confidence level was required, there is no scope for this with IPPC formulas (1) and (2). Only formulas (3) and (4) can be recalculated at different levels of confidence.

These points regarding the measure of variability and underlying probability distribution are reflected in the ratio of the margin of errors for the IPPC recommended formulas to formula (3). A ratio of one suggests the 2 formulas produce the same estimated infestation rate. The ratio is approximately equal to one (0.976) when there are 3 control samples or 4 control groups (0.950). Furthermore, the margin of error for IPPC formula (1) is double that of the lower one-sided 95% confidence limit (formula (3)) with just 6 individual control samples, and 5 times greater for 27 control samples. With 18 individual control samples as used in the tomato simulations, the ratio of the margin of errors is more than 4. The high ratios in the margin of errors show the potential for extreme conservatism in the estimated infestation rate when using the recommended IPPC formulas. This is particularly relevant when the control samples are held as individuals and larger sample numbers are employed. This will unconsciously inflate the true treatment efficacy by requiring an even larger number of samples to be treated to meet the requirements of the trading partner. Based on the estimated infestation rates for the mango and capsicum datasets, the recommended IPPC formulas would require 5 and 1.5 times, respectively, as many treated samples as estimated by the lower one-sided 95% confidence limit to meet the requirement of treating 30,000 or 93,616 target insects. This, in turn, will slow down the development of treatment schedules by consuming additional time and resources when they are not necessary.

The recommended calculations in the IPPC procedure manual also overlook the nature of the underlying distribution. Standard confidence intervals have a fundamental assumption, being that the sample distribution of the data is approximately normally distributed. Infestation rates may not always fulfill this assumption, with the data sometimes being positively skewed. This was observed for a proportion of the randomly generated subsets obtained from the tomato dataset. For 93.6% of the tomato subsets which were inconsistent with a normal distribution and did not contain a sample with no infestation recorded, the log-normal distribution could be assumed. Of the 5,000 randomly generated subsets, 19.7% were not consistent with the normal or log-normal distributions. In these cases, the most conceptually simple approach requires a data transformation, although it has been shown that applying a back-transformation can lead to misleading results (Pek et al. 2017). Confidence intervals can be formulated for some non-normal distributions, including the log-normal distribution as shown in formula (4). For other non-normal distributions, more sophisticated methods such as the parameter Wald method (Pek et al. 2017) and variations of the bootstrap method (Desharnais et al. 2015) could be considered. A level of discretion is required to allow researchers to use alternate methods for small non-normally distributed samples, although larger sample sizes and the use of the central limit theorem would avoid this issue.

When artificial infestation is applied, the calculation of the total number of treated insects is less problematic as a known number of insects are placed inside each sample. Artificial infestation has many advantages, but it is insufficient to simply assume treatment efficacy is not influenced by the infestation method. At present, there are very few publications available that compare artificial and natural infestation methods (Shellie and Mangan 2002, Hallman and Thomas 2010, 2011, Hallman 2014). The differences between artificial and natural infestation were not consistent in these publications, but they all highlight that the infestation method is an important consideration, and therefore, the issue of how to estimate the number of treated insects when using natural infestation needs to be addressed.

We have demonstrated that there are deficiencies in the statistical validity of the formulas currently recommended for calculating the treated infestation rate when using natural infestation. Based on the concerns outlined above, we are encouraging the quarantine research community and the IPPC to reconsider the currently recommended formulas for calculating the estimated infestation rate for phytosanitary treatment efficacy testing when the number of insects exposed to treatment is not known. For data that are approximately normally distributed, we recommend the lower one-sided confidence limit and for data approximately log-normal distributed, the lower one-sided modified Cox confidence limit is recommended. In the small number of situations where the data are inconsistent with the normal or log-normal distributions, there needs to be flexibility in the recommended approach to ensure the statistical validity of the resulting estimated infestation rate.
